# Fractalkine Signaling and Microglia Functions in the Developing Brain

**DOI:** 10.1155/2015/689404

**Published:** 2015-08-04

**Authors:** Isabelle Arnoux, Etienne Audinat

**Affiliations:** ^1^Institut National de la Santé et de la Recherche Médicale (INSERM), U1128, 75006 Paris, France; ^2^Université Paris Descartes, 75006 Paris, France; ^3^Focus Program Translational Neuroscience (FTN) and Institute for Microscopic Anatomy and Neurobiology, Johannes Gutenberg University Mainz, Hanns-Dieter-Hüsch-Weg 19, 55128 Mainz, Germany

## Abstract

Microglial cells are the resident macrophages of the central nervous system (CNS). Besides their classical roles in pathological conditions, these immune cells also dynamically interact with neurons and influence their structure and function in physiological conditions. The neuronal chemokine fractalkine and its microglial receptor CX3CR1 are one important signaling pathway involved in these reciprocal interactions. In the present review, we will discuss recent evidence indicating that fractalkine signaling also determines several functions of microglial cells during normal CNS development. It has been known for a decade that microglial cells influence the neuronal death that normally occurs during CNS development. Surprisingly, recent evidence indicates that they can also support survival of developing neurons, control axon outgrowth, and laminar positioning of subsets of interneurons in the forebrain. Moreover, microglial cells influence the maturation of synaptic circuits at early postnatal stages: their phagocytic activity allows them to eliminate inappropriate synapses and they can also influence the functional expression of synaptic proteins by releasing mediators. Fractalkine signaling controls these functions of microglial cells in part by regulating their timely recruitment at sites of developing synapses. Finally, on-going research suggests that this signaling pathway is also a key player in neurodevelopmental disorders.

## 1. Introduction

For a long time, microglial cells have been only studied for their roles in pathological conditions but the development of new genetic and functional analysis tools has started to reveal new functions of these resident immune cells of the central nervous system (CNS) [1–3].

Microglial cells are derived from myeloid precursors born in the yolk sac during primitive hematopoiesis which takes place at embryonic days (E) 7-E8 in mice. Microglia precursors then rapidly invade the brain where they are already detectable within the parenchyma at E9.5 [4–6]. Microglial cells are thus already present in the CNS when neurons migrate, proliferate, differentiate, and establish functional networks.

In the adult brain, microglial cells interact with neurons and synapses not only in pathological conditions but also in physiological conditions [7–10]. These interactions are controlled by several chemokine signaling pathways, including the fractalkine (or CX3CL1) signaling pathway [8]. In the CNS, fractalkine is mostly expressed by neurons and its unique receptor CX3CR1 is exclusively expressed by microglia [11]. Fractalkine is synthesized as a transmembrane protein containing 371 amino acid residues, consisting in a 76-amino acid N-terminal chemokine domain, a 241-amino acid glycosylated mucin-like stalk, a 18-amino acid hydrophobic transmembrane region, and a 37-amino acid intracellular C-terminal domain [11]. This protein can be cleaved by the lysosomal cysteine protease, cathepsin S, and members of the desintegrin and metalloproteinase (ADAM) family, such as ADAM-10 and ADAM-17, releasing a soluble form of fractalkine that contains the chemokine domain [12]. These two isoforms of fractalkine can interact with the microglial receptor CX3CR1, a G*α*i-coupled seven transmembrane domain receptor, the activation of which modulates several intracellular signaling pathways (PLC, PI3K, and ERK), and the recruitment of transcription factors (NF-kB, CREB) [12].

The fractalkine/CX3CR1 signaling pathway modulates microglial activation [13, 14]. In pathological conditions, microglial cells undergo important phenotypic changes to develop an adaptive response to the context [7]. This activation of microglial cells consists in modification of their morphology, proliferation, release of mediators, migration to the site of injury, and engulfment of cellular debris or dead cells [7, 8]. A large body of evidence indicates that constitutive expression of membrane-tethered fractalkine tends to inhibit microglia activation (off signal [15]). Accordingly, in several animal models of neuropathologies, including Parkinson disease, amyotrophic lateral sclerosis, stroke, and Alzheimer's disease, deficiency of fractalkine or of CX3CR1 leads to an increased production of proinflammatory molecules [12]. In particular interleukin-1*β* (IL-1*β*) and reactive oxygen species trigger a massive cell death [16–19]. However, in some of these pathological conditions, fractalkine/CX3CR1 signaling can also have neurotoxic effects and the inactivation of this signaling pathway precludes disease progression. In particular, in mouse models of Alzheimer disease, CX3CR1 deficiency induces a reduction of A*β* proteins accumulation due to the increase phagocytic activity of microglia [20]. In addition, in this particular disease, CX3CR1 deficiency decreases microglia activation and production of proinflammatory molecules such as IL-1*β*, tumor necrosis factor alpha (TNF*α*), and monocyte chemoattractant protein-1 (MCP-1 or CCL2) [20]. Similar results were obtained in fractalkine deficient mice for which, however, Tau phosphorylation is markedly increased [21]; see also [22]. Finally, CX3CR1 deficiency is also protective in cerebral ischemia [23–25]. Thus, neuroprotective and neurotoxic functions of the fractalkine/CX3CR1 signaling pathway are dependent on the microglial activation stimuli and pathological contexts.

In physiological conditions, recent evidence indicates that microglial cells contribute to the fine tuning of structural and functional properties of synaptic networks. Microglial cells continuously and dynamically survey their environment with their highly mobile processes [26, 27]. During this monitoring, microglia processes make transient contacts with synapses and the dynamics of these contacts is activity-dependent [28, 29]. It has been recently proposed that microglia process outgrowth toward synapses involves activation of neuronal NMDA receptors and dendritic release of ATP [30, 31]. Dynamics of basal motility of microglia process is regulated by fractalkine/CX3CR1 signaling. Confocal imaging of retinal explants has shown that the average velocity of spontaneous microglia process motility is lower in CX3CR1 deficient mice [32], suggesting that the neuronal chemokine fractalkine may regulate dynamics of microglial process motility and thus interactions between microglia and synapses.

Microglial cells in the adult CNS also shape adult hippocampal neurogenesis through apoptosis-coupled phagocytosis [33]. During adult neurogenesis in the subgranular zone of the dentate gyrus, only a small number of newborn cells survive and are integrated in preexisting circuits whereas the majority of newborn cells undergo apoptosis. Sierra and coworkers showed that microglial cells participate in the elimination of apoptotic newborn cells by phagocytosis. Interestingly, genetic disruption of CX3CR1 reduces cellular proliferation in the subgranular zone of the dentate gyrus, indicating that fractalkine/CX3CR1 signaling pathway promotes adult neurogenesis of the hippocampus [34, 35].

Finally, fractalkine/CX3CR1 signaling pathway influences also synaptic transmission in physiological conditions. Bath application of fractalkine transiently reduces the amplitude of AMPA receptor-mediated EPSCs in CA1 pyramidal neurons of the hippocampus* in vitro* [36] but enhances the amplitude of the NMDA receptor-mediated component of these EPSCs [37]. Interestingly, fractalkine expression is upregulated in the hippocampus during memory-associated synaptic plasticity [38]. Yet, the consequence of CX3CR1 disruption on long-term synaptic potentiation (LTP) in the hippocampus remains controversial, one group reporting an inhibition of LTP [35], whereas another reported an increased LTP [39] in CX3CR1 deficient mice.

Thus, fractalkine/CX3CR1 signaling governs several functions of microglial cells in the adult brain in pathological but also in physiological conditions. In the following sections we will provide an overview of fractalkine/CX3CR1 signaling implication in the development of the CNS, highlighting key functions of microglia in building up neuronal and synaptic networks during development.

## 2. Fractalkine Signaling and Neuronal Survival during Development

**Figure 1:**
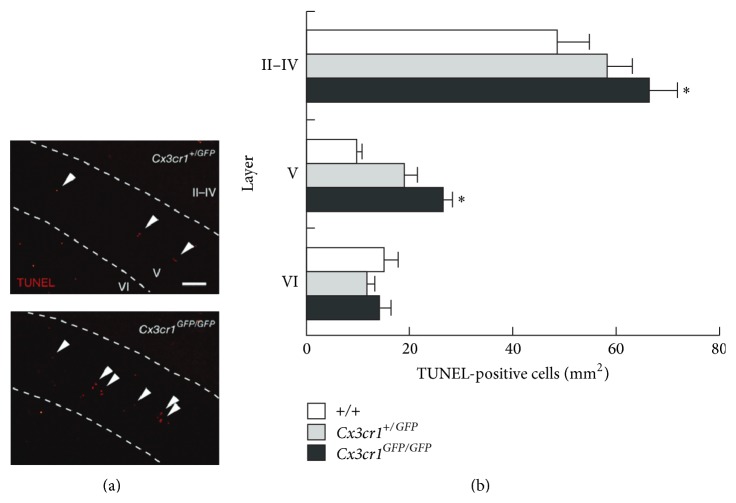
Fractalkine/CX3CR1 signaling controls survival of cortical neuron during early postnatal development. (a) Apoptotic cells revealed by TUNEL staining in the cortex of* Cx3cr1* ^*+/GFP*^ (top) and* Cx3cr1* ^*GFP/GFP*^ (bottom) mice at P5. Scale bar represents 100 *μ*m. The arrowheads indicate TUNEL-positive elements in the layer V of the cortex. (b) Quantification of the TUNEL-positive cells density in different cortical layers. Note the increase of apoptotic cells in the layer V and II–IV in the cortex of* Cx3cr1* ^*GFP/GFP*^. Adapted from [43].

It has been known for long that microglial cells are involved in the induction of the neuronal death that normally occurs during CNS development (for review [40]). However, microglia can also promote neuronal survival in the developing postnatal forebrain. During CNS development, neurons require trophic support to survive and to be integrated in neuronal circuits. In the subventricular zone, microglial cells promote neurogenesis and oligodendrogenesis trough the release of proinflammatory molecules such as IL-1*β*, IL-6, TNF-*α*, and IFN-*γ* [41]. The use of minocycline, a classical inhibitor of microglia activation, to challenge pharmacologically microglial functions induces a decrease in levels of a number of cytokines and the inhibition of neurogenesis and oligodendrogenesis. It should be pointed out, however, that minocycline should be used with cautious in the developing CNS where it can lead to paradoxical activation of microglia [42]. Microglial cells were also shown to promote survival of layer 5 pyramidal neurons of the motor cortex during the first postnatal week [43]. Minocycline treatment but also transient ablation of microglial cells leads to the apoptosis of layer 5 neurons projecting to subcortical targets or to the contralateral cortex. Evidence from* in vivo* and* in vitro* experiments further indicates that microglial cells located on the trajectory of layer 5 axons provided a trophic effect through the release of insulin growth factor-1 (IGF-1). Surprisingly, CX3CR1 deficiency which increases the number of microglia present in the subcortical white matter also impairs this trophic action of microglia and leads to increased apoptosis of layer 5 neurons (Figure 1). This impairment of the trophic role of microglia in CX3CR1 deficient mice could result from an upregulation of IGF-1 binding proteins that bind IGF-1 to inhibit its trophic functions [43]. Therefore, the production of factors by microglia is essential to promote cell survival during postnatal development and fractalkine signaling regulates this function of microglia.

## 3. Fractalkine Signaling and Microglia Recruitment in Developing CNS Structures

**Figure 2:**
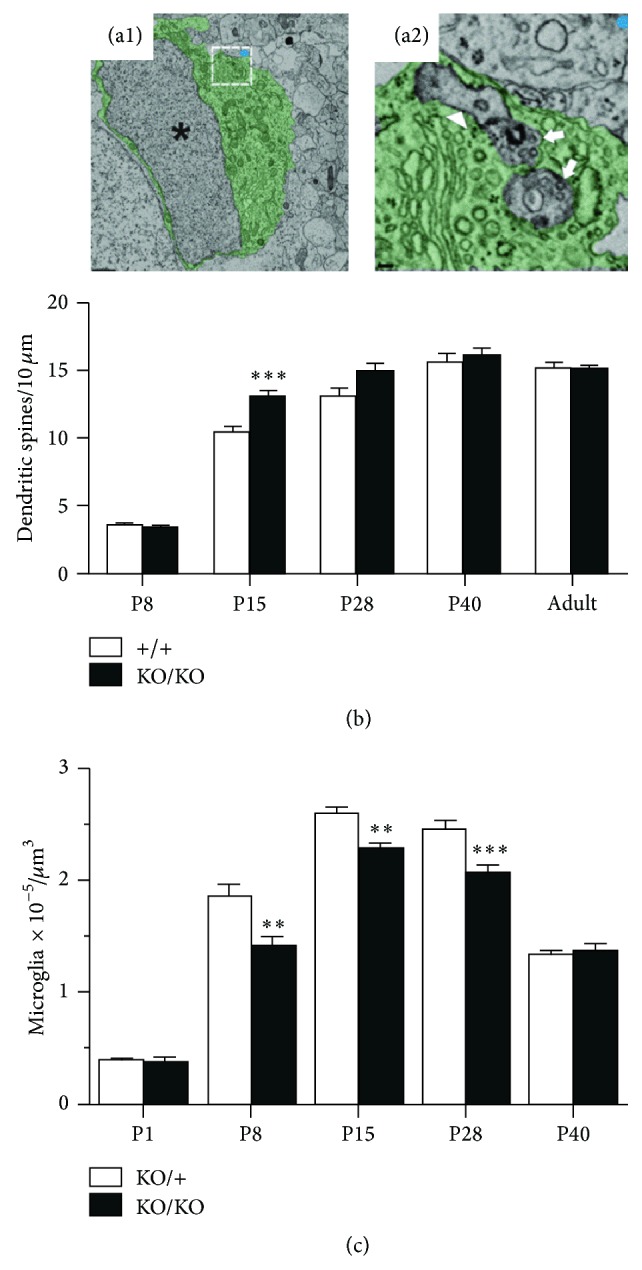
Fractalkine/CX3CR1 signaling modulates synaptic pruning by microglia during postnatal development. (a) Microglial cells remove presynaptic elements by synaptic pruning at P5 in the retinogeniculate system. (a1) Low magnification electronic microscopy of microglia. Asterisks denote the nucleus and the cytoplasm is pseudocolored green. Scale bar = 1 *μ*m. (a2) Magnified regions of (a1) (white box) demonstrating membrane-bound elements engulfed by microglia. Arrows designate elements containing presynaptic machinery (40 nm vesicles). The arrowhead designates engulfed material resembling juxtaposed postsynaptic elements. Scale bar = 100 nm. Adapted from [53]. (b) A transient increase in dendritic spine density was observed in CX3CR1 deficient (KO/KO) mice when compared with WT (+/+) littermates during the second postnatal week (^*∗∗∗*^ *p* < 0.0001). This transient increase in dendritic spines number could result of a transient deficit of synaptic pruning. (c) Quantification of microglia nuclei in the CA1 stratum radiatum from the hippocampus revealed a transient decrease in microglia density in CX3CR1 deficient mice at P8, P15, and P28 compared with control littermates (^*∗∗*^ *p* < 0.005). This decrease in microglia number in KO mice suggests a transient delayed microglia recruitment which can explain the transient deficit of synaptic pruning. ((b) and (c)) Adapted from [47].

**Figure 3:**
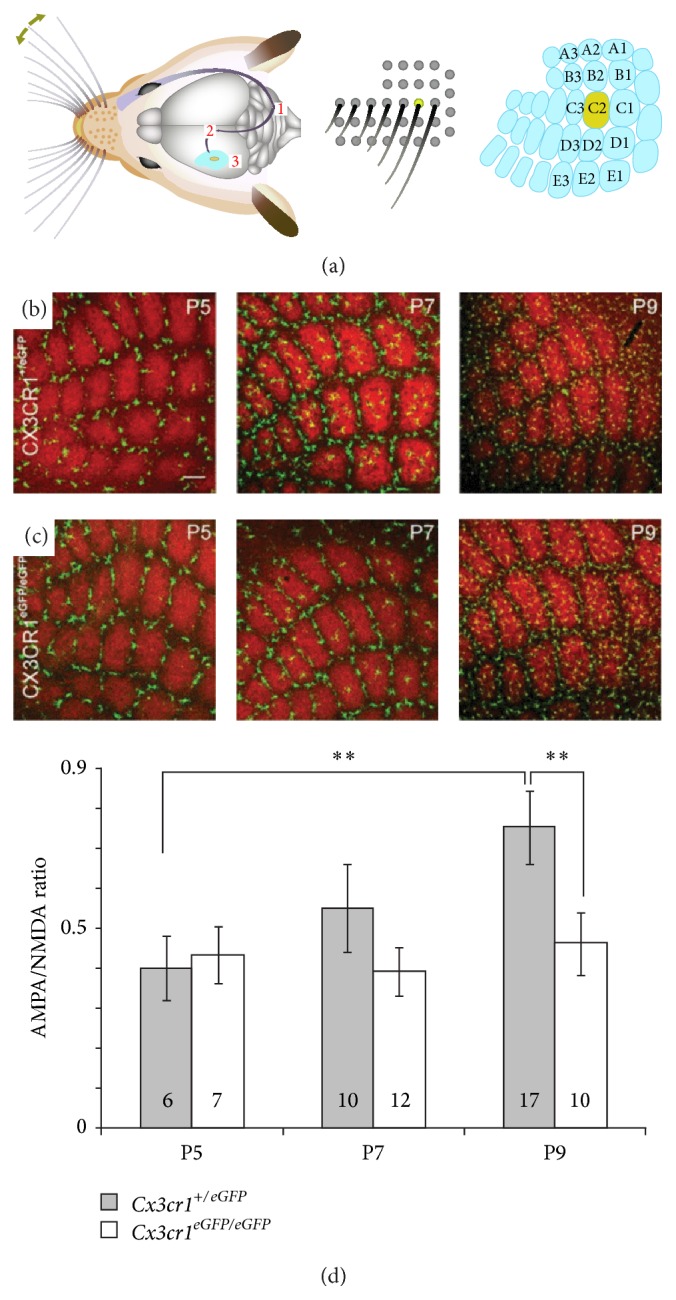
Fractalkine/CX3CR1 signaling controls the recruitment of microglia and the functional maturation of thalamocortical synapses. (a) Drawing of the sensory system of vibrissae in rodents and link between the distribution of vibrissae and that of barrels in layer 4 somatosensory cortex. Adapted from [59]. ((b) and (c)) Microglia (green) distribution in the layer 4 of the somatosensory cortex during postnatal development in* Cx3cr1* ^*+/eGFP*^ (b) and* Cx3cr1* ^*eGFP/eGFP*^ (c) mice. At P5, microglial cells are exclusively located outside of the barrel centers which contain thalamocortical synapses (red, staining of thalamocortical axons). At P7, microglial cells start to invade the barrel centers in* Cx3cr1* ^*+/eGFP*^ mice and this invasion is delayed in* Cx3cr1* ^*eGFP/eGFP*^ mice. At P9, microglial cell distribution is similar for the two genotypes. Scale bar, 100 *µ*m. (d) Relative change in the synaptic currents resulting of the activation of AMPA (*α*-amino-3-hydroxy-5-methyl-4-isoxazolepropionic acid) and NMDA (N-methyl-D-aspartate) postsynaptic receptors expressed at thalamocortical synapses between P5 and P9 in* Cx3cr1* ^*+/eGFP*^ and* Cx3cr1* ^*eGFP/eGFP*^ mice. Note that the AMPA/NMDA ratio increases between P5 and P9 in* Cx3cr1* ^*+/eGFP*^ but not in* Cx3cr1* ^*eGFP/eGFP*^ mice. ((b) and (c)) Adapted from [48].

**Figure 4:**
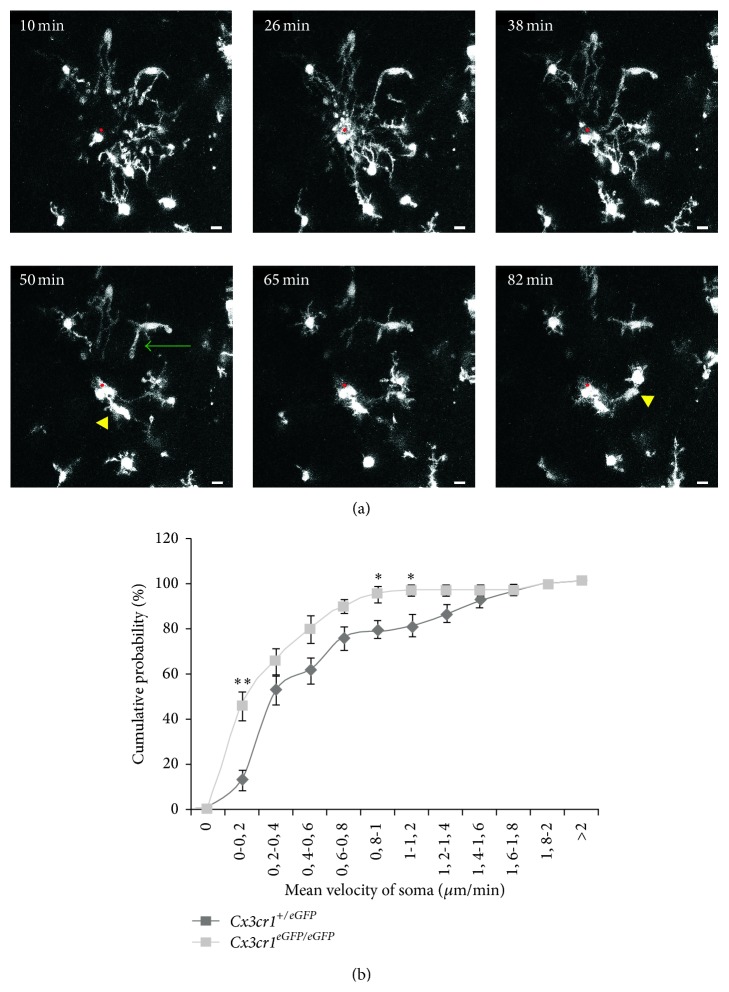
Impaired microglia motility in developing CX3CR1 deficient mice. (a) Two-photon images in an acute slice of a P7* Cx3cr1* ^*+/eGFP*^ mouse showing the dynamics of microglial processes and soma after the insertion (at time 0 min, not shown) of a pipette (red dot) containing 2-MeSADP (100 *µ*M). Yellow arrowheads indicate the soma of 2 microglial cells moving toward the pipette. Green arrow indicates a retracting process. Calibration bar is 10 *µ*m. (b) Comparison of the mean velocity of microglia soma toward the 2-MeSADP-containing pipette for* Cx3cr1* ^*+/eGFP*^ (50 cells, 6 experiments)* Cx3cr1* ^*+/eGFP*^ (42 cells, 5 experiments) animals (^*∗*^ *p* < 0.05, ^*∗∗*^ *p* < 0.01, Mann-Whitney test).

In adult rodents, there is a rather high density and a homogeneous distribution of microglial cells throughout the CNS parenchyma. In contrast, the embryonic and early postnatal CNS is characterized by a low density and a highly heterogeneous distribution of microglia [44, 45]. Several lines of evidence indicate that fractalkine/CX3CR1 signaling is involved in the timely recruitment of microglial cells at specific locations. In the embryonic spinal cord, for instance, microglial cells aggregate at embryonic day (E) 12.5 in the dorsolateral region close to terminals of dying dorsal root ganglia neurons and at E13.5 in the ventral region within lateral motor columns where motoneurons start to undergo developmental cell death [46]. In the embryonic telencephalon, microglial cells are transiently associated with the extremities of midbrain dopaminergic axons as they enter the subpallium, but not with adjacent serotonin or internal capsule fibers [45]. Phagocytic microglial activity at this point seems to restrain dopaminergic fiber extension and, remarkably, this action of developing microglia is impaired in CX3CR1 deficient mice [45]. One reason that could explain the role of fractalkine signaling in modulating dopaminergic fiber extension is the control of microglia recruitment by this specific signaling pathway during CNS development. During the second and the third postnatal weeks, there is, transiently, a reduced microglia number in the hippocampus of CX3CR1 deficient mice (Figure 2(c) and [47]), suggesting that fractalkine/CX3CR1 controls the timing of microglia colonization of CNS parenchyma. However, this colonization is probably determined by the recruitment of microglia at maturating synapses [48]. In the developing layer 4 of the somatosensory “barrel” cortex, microglial cells remain outside the areas containing the maturating thalamocortical synapses (i.e., the barrel centers) until P5 and colonize these areas between P6 and P9 (Figure 3(b)). A similar pattern of microglia distribution and recruitment was observed during the postnatal development of the olfactory bulb around glomeruli which are also areas of high synapse density [49]. Interestingly, fractalkine immunoreactivity is transiently increased between P5 and P10 within the barrel centers [48] and the colonization of the barrel centers is delayed by 2-3 days in CX3CR1 deficient mice (Figure 3(c)), despite the fact that the overall density of microglial cells within layer 4 is not affected in mutant mice [48]. Moreover, recruitment of microglial cells in the barrels centers is associated with a transient expression of a specific phenotype of layer 4 microglia and the acquisition of this phenotype is also delayed by 2-3 days in CX3CR1 deficient mice [44]. This suggests that fractalkine signaling favors the attraction of microglia by maturating synapses. Because CX3CR1 deficiency decreases microglia migration (toward lesion sites) in the retina of young adult mice [32], we tested whether microglial cell motility was also impaired in the developing barrel cortex of CX3CR1 deficient mice. Using two-photon microscopy in acute slices of P5–P9* Cxr3cr1* ^*+/eGFP*^ or* Cx3cr1* ^*eGFP/eGFP*^ mice, we followed microglial cell motility when a pipette containing the P2Y12 receptor agonist, 2Me-S-ADP (100 *µ*M), was introduced in acute cortical slices. P2Y12 receptors govern microglia motility and chemotaxis in response to nucleotides [50]. As this is the case in slices of adult mice [51], microglial cells start by sending their processes toward the point source of 2-Me-S-ADP (Figure 4(a)). In marked contrast with the situation in adult slices, however, we observed that a significant number of developing microglial cells retracted their processes after having reached the pipette, whereas others translocated their nucleus within a leading process targeting the pipette tip (Figure 4(a)). Quantification of the soma velocity indicated that microglial cells moved toward the source of P2Y12 receptor agonist at lower speed in* Cx3cr1* ^*eGFP/eGFP*^ than in* Cx3cr1* ^*+/eGFP*^ mice (Figure 4(b)). Thus, by favoring microglia attraction at synaptic sites and by modulating microglia motility, CX3CL1/R1 signaling may play a major role in determining how microglial cells influence the maturation of synaptic circuits.

## 4. Fractalkine Signaling and Maturation of Synaptic Circuits

During initial steps of the development of neuronal network, there is an overproduction of synaptic contacts (reviewed in [52]). Mature networks are then formed through activity-dependent mechanisms leading to the elimination of weak synapses and functional maturation of the remaining ones. Recent evidence indicates that microglial cells are involved in the elimination of supernumerary synapses during development. In particular, Schafer et al. (2012) demonstrated that, during postnatal development of the dorsolateral geniculate nucleus of the thalamus, microglia can engulf synaptic elements (Figure 2(a)), in an activity-dependent manner and through a microglia-specific phagocytic signaling pathway involving the C3 component of the complement cascade and its microglial receptor of C3 (CR3). The pruning of weak C3 tagged synapses by microglia participate in the refinement of neuronal connectivity allowing the appropriate segregation of ipsi and contralateral retinogeniculate terminals in the thalamus [53].

This synaptic pruning by microglia has been also observed in the hippocampus where this developmental process is regulated by fractalkine/CX3CR1 signaling [47]. STED and electron microscopy revealed the presence of synaptic material engulfed by microglial processes in the hippocampus during the first postnatal weeks. Comparative analysis of wild type and CX3CR1 deficient mice indicates that CX3CR1 deficiency is associated with a higher number of dendritic spines (Figure 2(b)), a higher density of PSD95 immunoreactive puncta, and impaired functional properties of the hippocampal excitatory synaptic network during postnatal development. These observations could be explained by a deficit in synaptic pruning due to the delayed recruitment of microglial cells in the developing hippocampus of CX3CR1 deficient mice (see above). Interestingly, the number of spines and the density of microglial cells in the hippocampus in these mice eventually match those of wild type animals after the end of the first postnatal month [47]. Yet, CX3CR1 deficient adult mice have weak synaptic transmission, decreased functional brain connectivity, deficits in social interactions, and increased repetitive-behavior phenotypes [54]. Impaired synaptic pruning probably contributes but is unlikely the only cause of this adult phenotype since, for instance, CX3CR1 deficiency also leads to impaired long-term potentiation and decreased survival and proliferation of adult neural progenitors due to an excess of IL-1*β* production [34, 35].

In the developing barrel cortex, the delayed recruitment of microglia in the barrel centers is associated with a transient impairment of the functional maturation of thalamocortical synapses: the increase of the ratio of AMPA/NMDA receptors (Figure 3(c)) and the switch of GluN2B to GluN2A NMDA receptor subunits which normally occurs at thalamocortical synapses around the end of the first postnatal week are both impaired in CX3CR1 deficient mice. However, the same functional parameters of thalamocortical synapses do not differ between adult CX3CR1 deficient and wild type mice [48] (Arnoux & Audinat, unpublished). These observations suggest that the presence of microglia within the barrel centers is necessary for the functional maturation of thalamocortical synapses during postnatal development. The exact mechanism by which microglial cells influence this maturation remains to be identified. Several signaling molecules known to be released by microglia are also known to modulate the functional expression of glutamatergic receptors. For instance, TNF*α* of glial origin is known to increase AMPA receptors trafficking and membrane insertion [55, 56]. Similarly, brain-derived neurotrophic factor (BDNF) released by microglia has been shown to modulate spine density but also the expression of AMPA and NMDA receptors in cortical neurons of adult mice [57]. Thus, fractalkine-dependent recruitment of microglial cells within the barrel centers may allow the secretion of microglia-derived signaling molecules necessary for inducing changes in the functional expression of glutamate receptors at thalamocortical synapses.

## 5. Conclusions

It is now clear that reciprocal interactions between neurons and microglia contribute to the physiological development of the CNS. The fractalkine/CX3CR1 signaling pathway regulates these interactions partly by controlling microglia recruitment at specific sites but also by influencing the phenotype and thus the functions of these immune cells during development. Considering also the importance of this signaling pathway in pathological conditions in adulthood [8], fractalkine/CX3CR1 signaling is likely to be a key actor of neurodevelopment disorders. Interestingly, CX3CR1 deficient mice have been shown recently to have deficits in social interaction and increased repetitive-behavior phenotypes that have been previously associated with neurodevelopmental and neuropsychiatric disorders [54]. From this point of view and independently of fractalkine/CX3CR1 signaling, it is worth noting that dysfunction of microglial cells is increasingly suspected to occur in psychiatric diseases associated with neurodevelopmental disorders (reviewed in [58]). We are thus at the beginning of an exciting time for the study of microglia functions during normal and pathological development and these extremely plastic cells have not yet finished to reveal their multiple facets.
